# Tumour‐induced alterations in single‐nucleus transcriptome of atrophying muscles indicate enhanced protein degradation and reduced oxidative metabolism

**DOI:** 10.1002/jcsm.13540

**Published:** 2024-07-13

**Authors:** Samet Agca, Aylin Domaniku‐Waraich, Sevval Nur Bilgic, Melis Sucuoglu, Meric Dag, Sukru Anil Dogan, Serkan Kir

**Affiliations:** ^1^ Department of Molecular Biology and Genetics Koç University Istanbul 34450 Turkey; ^2^ Department of Molecular Biology and Genetics, Center for Life Sciences and Technologies Boğaziçi University Istanbul Turkey

**Keywords:** cancer cachexia, EDA2R signalling, single‐nucleus RNA sequencing, skeletal muscle atrophy

## Abstract

**Background:**

Tumour‐induced skeletal muscle wasting in the context of cancer cachexia is a condition with profound implications for patient survival. The loss of muscle mass is a significant clinical obstacle and is linked to reduced tolerance to chemotherapy and increased frailty. Understanding the molecular mechanisms driving muscle atrophy is crucial for the design of new therapeutics.

**Methods:**

Lewis lung carcinoma tumours were utilized to induce cachexia and muscle atrophy in mice. Single‐nucleus libraries of the tibialis anterior (TA) muscle from tumour‐bearing mice and their non‐tumour‐bearing controls were constructed using 10X Genomics applications following the manufacturer's guidelines. RNA sequencing results were analysed with Cell Ranger software and the Seurat R package. Oxygen consumption of mitochondria isolated from TA muscle was measured using an Oroboros O2k‐FluoRespirometer. Mouse primary myotubes were treated with a recombinant ectodysplasin A2 (EDA‐A2) protein to activate EDA‐A2 receptor (EDA2R) signalling and study changes in gene expression and oxygen consumption.

**Results:**

Tumour‐bearing mice were sacrificed while exhibiting moderate cachexia. Average TA muscle weight was reduced by 11% (*P* = 0.0207) in these mice. A total of 12 335 nuclei, comprising 6422 nuclei from the control group and 5892 nuclei from atrophying muscles, were studied. The analysis of single‐nucleus transcriptomes identified distinct myonuclear gene signatures and a shift towards type IIb myonuclei. Muscle atrophy‐related genes, including *Atrogin1*, *MuRF1* and *Eda2r*, were upregulated in these myonuclei, emphasizing their crucial roles in muscle wasting. Gene set enrichment analysis demonstrated that EDA2R activation and tumour inoculation led to similar expression patterns in muscle cells, including the stimulation of nuclear factor‐kappa B, Janus kinase–signal transducer and activator of transcription and transforming growth factor‐beta pathways and the suppression of myogenesis and oxidative phosphorylation. Muscle oxidative metabolism was suppressed by both tumours and EDA2R activation.

**Conclusions:**

This study identified tumour‐induced transcriptional changes in muscle tissue at single‐nucleus resolution and highlighted the negative impact of tumours on oxidative metabolism. These findings contribute to a deeper understanding of the molecular mechanisms underlying muscle wasting.

## Introduction

Tumour‐induced skeletal muscle wasting is one of the characteristics of cancer cachexia, a deadly condition that negatively influences the prognosis and the survival of patients. Progressive weight loss due to the wasting of muscle and adipose tissues affects the majority of patients with gastric, pancreatic, lung, colorectal and prostate cancers and accounts for at least 20% of all cancer deaths.^1^ Muscle wasting is associated with frailty, chemotherapy intolerance and a lack of response to treatment.^2^ Loss of muscle mass results in impaired physical strength and the consequent poor quality of life, which is often irreversible due to a lack of effective therapeutics.^3^ Wasting is driven by the atrophy of muscle tissue, which involves reduced protein synthesis and excessive proteolysis.^4^ Despite the progress made in understanding the molecular mechanisms underlying muscle atrophy, additional research is needed to uncover novel players and develop new tools for treatment.

Our recent work demonstrated that ectodysplasin A2 (EDA‐A2) receptor (EDA2R) signalling mediates tumour‐induced muscle wasting by enhancing the expression of muscle atrophy‐related genes, including *Atrogin1* and *MuRF1*.^5^ EDA2R is a member of the tumour necrosis factor (TNF) receptor superfamily.^6^ The expression of EDA2R and its ligand, EDA‐A2, is highly enriched in skeletal muscle tissue.^5^ The overexpression of EDA‐A2 in muscle tissue was found to cause severe myodegeneration.^7^ We have shown that cachexia‐associated muscle atrophy is accompanied by the upregulation of EDA2R expression,^5^ which was originally identified using whole transcriptome analysis. Although bulk transcriptomics is instrumental in studying disease states in different tissues, a major limitation of this technique is that the expression profiles of a heterogeneous cell population are averaged, and valuable information on differential gene expression in specific subsets of the cells is lost. In this study, we utilized high‐throughput, high‐resolution transcriptomics to determine tumour‐induced alterations in muscle gene expression at single‐nucleus resolution.

Skeletal muscle tissue is composed of giant syncytial cells called myofibers, each containing numerous myonuclei. While single‐cell RNA sequencing (scRNA‐seq) enabled studying mononucleated cells in muscle tissue,^8^ high‐throughput profiling of both mononuclear and multinuclear cells is achieved by single‐nucleus (sn)RNA‐seq. This technique involves nuclear isolation and detects nuclear‐enriched transcripts, including pre‐mRNA.^9^, ^10^ snRNA‐seq studies on skeletal muscle tissues indicated that *Titin*‐expressing myonuclei account for the majority of the nuclei (~65%), while the remaining originate from mononuclear cells, including muscle satellite cells (MuSCs), fibro‐adipogenic progenitors (FAPs), endothelial cells, tenocytes, smooth muscle cells and immune cells.^9^, ^10^, ^11^, ^12^, ^13^, ^14^, ^15^, ^16^, ^17^, ^18^ Adult skeletal muscle comprises fast‐ and slow‐twitch myofibers classified by the expression of specific myosin heavy chain (MYH) genes. In mice, slow myofibers are enriched in *Myh7* expression (type I), while fast myofiber types express high levels of *Myh2* (type IIa), *Myh1* (type IIx) or *Myh4* (type IIb). snRNA‐seq analysis enables the detection of distinct myonuclear gene signatures representing myofiber types and specialized functions, such as the neuromuscular junction (NMJ) and the myotendinous junction (MTJ).^19^, ^20^ Here, we report that remote tumour growth in mice impacted the transcriptome of muscle tissue, which exhibited the enrichment of type IIb myonuclear gene signatures, the activation of muscle atrophy‐related mechanisms, including EDA2R, and the suppression of pathways associated with muscle contractility and oxidative metabolism.

## Materials and methods

### Mice

Mice were housed at 22°C and under 50% humidity with 12 h of light and 12 h of dark cycles (07:00–19:00) and given ad libitum access to a standard rodent chow diet and water in the Koç University Animal Research Facility in accordance with institutional policies and animal care ethics guidelines. Eight‐ to 12‐week‐old male mice with a C57BL/6 background were used for the experiments. EDA2R knockout mice were previously described.^5^, ^7^ Lewis lung carcinoma (LLC) cells were used for tumour inoculation. LLC cells were cultured in Dulbecco's modified Eagle's medium (DMEM) (Sigma, No. 5796) with 10% foetal bovine serum (FBS) and penicillin/streptomycin (PS) (Invitrogen). A total of 5 × 10^6^ LLC cells were injected subcutaneously over the flank, while control mice received phosphate‐buffered saline (PBS) only. Tibialis anterior (TA) muscles contralateral to the tumour inoculation site were harvested 16 days after LLC inoculation. In each experiment, different cohorts of mice were utilized, and TA samples were used as whole pieces.

### Tissue histology

Isopentane was cooled with liquid nitrogen until it was at least half‐frozen. TA muscle samples were placed in the cooled isopentane for 10 s. The frozen tissues were embedded in Tissue‐Tek OCT freezing medium (Sakura) and cut into 8‐μm‐thick sections using a cryostat. Sections were collected on Superfrost Plus slides (Thermo). The sections were fixed with 4% paraformaldehyde and stained with haematoxylin (Merck, No. 105174), 0.1% hydrochloric acid, eosin (Merck, No. 109844), a 70–100% ethanol gradient and xylene (Isolab). The cross‐sectional area of the muscle fibres was measured using ImageJ software.

### Immunofluorescence staining of muscle sections

Cryosections of the TA muscles were incubated with a blocking solution (3% bovine serum albumin [BSA] + 0.1% Triton X‐100 + 5% horse serum) for 1 h at room temperature and then with monoclonal anti‐myosin heavy chain (MyHC) antibodies in the blocking solution overnight at +4°C. MyHC antibodies (DSHB) were as follows: SC‐71 (IgG1, 1:100 dilution) specific for MyHC‐2A; BF‐F3 (IgM, 1:100 dilution) specific for MyHC‐2B; 6H1 (IgM, 1:50 dilution) specific for MyHC‐2X; and BA‐F8 (IgG2b, 1:50 dilution) specific for MyHC‐I (slow, alpha and beta fibres). Sections were washed with PBS and incubated in the blocking solution containing goat anti‐mouse IgM Alexa Fluor 488 (Abcam, ab150121, 1:500) antibody and goat anti‐mouse IgG H&L Alexa Fluor 594 (Abcam, ab150116, 1:500) antibody for 1 h. Finally, the sections were mounted using a homemade mounting medium. Using fluorescence microscopy tile imaging (Zeiss), whole TA cross‐sections were acquired, which were analysed for fibre type composition and cross‐sectional area with ImageJ software.

### Primary myoblast culture

Mouse primary myoblasts were isolated from the limb muscles of 2‐ to 3‐day‐old pups and cultured in Ham's F‐10 nutrient mixture (Invitrogen) as described before.^21^ The culture medium was supplemented with 20% FBS (Invitrogen), 2.5 ng/mL of basic fibroblast growth factor (bFGF; Sigma) and PS (Invitrogen). For differentiation, cultured myoblast cells were transferred to DMEM (Sigma 5796) with 5% horse serum (Invitrogen) and PS. Myoblasts differentiated into myotubes were treated with 250 ng/mL recombinant EDA‐A2 protein (R&D Systems 922‐ED) for 24 or 48 h.

### Immunofluorescence staining of primary myotubes

Cells fixed in ice‐cold 100% methanol at −20°C for 10 min were incubated in a blocking solution containing 3% BSA, 0.1% Triton X‐100 and 10% horse serum at room temperature for 1 h. Cells were then incubated with a MyHC antibody (DSHB, MF20) at a 1:1000 dilution in the blocking solution for 1 h at room temperature. After washing with PBS, the cells were treated with an anti‐mouse IgG H&L Alexa Fluor 594 secondary antibody (Abcam, ab150116) at a 1:2000 dilution and DAPI (Cayman) at a 1:3000 dilution in the blocking solution for 1 h. Cells mounted with a homemade medium were visualized using a Zeiss fluorescence microscope. At least 10 distinct myotubes were analysed for each image. The diameters of individual myotubes were measured at three different sites with values averaged using ImageJ software.

### RT‐qPCR

Total RNA from cultured myotubes or TA muscle samples was extracted using Qiazol reagent (Qiagen), and purification was performed using RNA spin columns (Ecotech). TissueLyzer LT (Qiagen) was used to homogenize the tissues. A high‐capacity cDNA reverse transcription kit (Thermo) was used for complementary DNA synthesis. Reactions were set with 25 ng of cDNA, 150 nmol of specific primers and iTaq Universal SYBR Green Supermix (Bio‐Rad). Gene expression was analysed by RT‐qPCR using a CFX Connect instrument (Bio‐Rad). Calculations for relative mRNA levels were done by the ΔΔCt method and normalized to cyclophilin mRNA.

### Bulk RNA sequencing

Mouse primary myoblasts were differentiated into myotubes for 48 h and then treated with 250 ng/mL recombinant EDA‐A2 for 24 h. Isolation and DNase treatment of total RNA were performed using Qiazol reagent (Qiagen) and the Direct‐zol RNA MiniPrep kit (Zymo Research). Isolated samples were prepared with the Illumina TruSeq Stranded mRNA Library Prep Kit. Illumina NovaSeq technology was used for pair‐end sequencing, which produced a total of 60 million (2 × 100 bp) paired‐end reads per sample. The quality check of raw sequencing data was done with FastQC (v0.11.9), and reports were created with MultiQC (v1.12). Trimming of poor‐quality reads and adaptor sequences was performed with trimGalore (v0.6.5). A mouse genome index (GRCm39) was created, and reads were mapped using STAR (v2.7.3a). The quality of the mapped data was checked with qualimap (v2.2.1). Quantifications of alignments were done by HTSeq (v0.11.1).

### Skeletal muscle nuclei isolation

A previously described protocol was used to isolate nuclei.^11^ Briefly, TA muscle samples dissected from six mice in each group were combined and processed together. Samples were chopped with dissection scissors and placed into a lysis buffer containing 10 mM Tris–HCl, 10 mM NaCl, 3 mM MgCl_2_ and 0.1% NP40 in nuclease‐free water. Samples were homogenized using a douncer and filtered with 70 and 40 μm of cell strainers. After centrifugation for 5 min at 500 g at 4°C, the supernatant was discarded, and the nuclei were resuspended, stained with DAPI and subjected to fluorescence‐activated cell sorting.

### Single‐nucleus RNA sequencing and bioinformatic analyses

10X Genomics applications were used following the manufacturer's guidelines (Chromium Next GEM Single Cell 3′ Reagent Kits) to prepare the libraries, which were sequenced using the Illumina HiSeq X system. The sequencing strategy was designed to detect 7000 nuclei per condition and 50 000 reads per nucleus. Sequencing data were first analysed and filtered using the Cell Ranger (v7.0) Single‐Cell Software Suite provided by 10X Genomics, which yielded 6622 nuclei for the control group and 5998 nuclei for the cachectic group. Data were counted and mapped using the cellranger count function with "include introns" option for pre‐mRNAs. Further analysis was performed using the Seurat (v4.3.0) R (v4.2.2) package on RStudio (v2022.12.0), which filters nuclei, normalizes expression data and carries out principal component analysis for clustering and uniform manifold approximation and projection (UMAP) visualization. After the filtering step, which removes damaged nuclei (with <200 genes or >1% mitochondrial RNA) or duplicate nuclei (with >5000 genes), we obtained 6422 nuclei for the control group and 5892 nuclei for the cachectic group. The Seurat R package was also used to perform marker assignment of the clusters and gene ontology analysis of their uniquely expressed genes. ClusterProfiler (4.7.1.003) R package was used for Kyoto Encyclopedia of Genes and Genomes (KEGG) pathway enrichment analysis, and gene set enrichment analysis (GSEA) software (v4.3.2) was used for hallmark gene sets enrichment analysis. The Monocle3 analysis toolkit was used for single‐cell trajectory analysis. This algorithm identifies branching trajectories based on gene expression similarities.

### Mitochondria isolation, oxygen consumption and H_2_O_2_ production rate measurements

Mitochondria were isolated from the TA muscle. Following dissection, the muscles were cut into small pieces and incubated in ice‐cold PBS supplemented with 10 mM EDTA and 0.05% trypsin for 30 min. The supernatant was discarded after centrifugation at 200 g for 5 min at 4°C. The pellet was resuspended in IB_m_1 buffer (67 mM sucrose, 50 mM KCl, 50 mM Tris–HCl, 10 mM EDTA, 0.2% fatty acid‐free BSA, pH 7.4) and homogenized using a Teflon‐Glass pestle. The homogenous solution was centrifuged at 700 g for 10 min at 4°C. The supernatant was transferred into a new tube and centrifuged at 8000 g for 10 min at 4°C. The pellet containing mitochondria was resuspended in 50 μL of IB_m_2 buffer (250 mM sucrose, 0.3 mM EGTA‐Tris, 10 mM Tris–HCl, pH 7.4).

Oxygen consumption rates were measured using an Oroboros O2k‐FluoRespirometer (Oroboros Instruments, Innsbruck, Austria). A total of 130 μg of muscle mitochondria was diluted in 2 mL of mitochondrial respiration buffer MiR05 (0.5 mM EGTA, 3 mM MgCl_2_, 60 mM lactobionic acid, 20 mM taurine, 10 mM KH_2_PO_4_, 20 mM HEPES, 110 mM sucrose, 1 mg/mL fatty acid‐free BSA, pH 7.2) inside the Oroboros O2k‐FluoRespirometer chambers. A substrate‐uncoupler‐inhibitor‐titration (SUIT) protocol was used to determine the additive effect of Complex I‐ and Complex II‐linked substrates in isolated mitochondria. The following chemicals are added in order: 10 mM glutamate, 2 mM malate, 2.5 mM adenosine diphosphate (ADP), 10 mM succinate, 0.5 μM rotenone, 10 nM oligomycin, stepwise 0.5 μM carbonyl cyanide 3‐chlorophenylhydrazone (CCCP) and 2.5 μM antimycin A.

A total of 3 × 10^5^ primary myotubes per well were incubated with their culture medium in the Oroboros O2k‐FluoRespirometer. Routine respiration represented the physiological coupling state of the myotubes; the ‘Electron Transfer (ET) capacity’ was obtained by the stepwise addition of 0.5 μM CCCP.

H_2_O_2_ production rate was measured at 37°C using 130 μg of muscle mitochondria diluted in 2 mL of mitochondrial respiration buffer MiR05 (0.5 mM EGTA, 3 mM MgCl_2_, 60 mM lactobionic acid, 20 mM taurine, 10 mM KH_2_PO_4_, 20 mM HEPES, 110 mM sucrose, 1 mg/mL fatty acid‐free BSA, pH 7.2) in an Oroboros O2k‐FluoRespirometer. The reaction of H_2_O_2_ and Amplex® UltraRed (10 μM) is catalysed by horseradish peroxidase (1 U/mL) and superoxide dismutase (5 U/mL) to produce the red fluorescent compound resorufin. The reaction is initiated by the addition of succinate (10 mM), followed by 0.5 μM rotenone, 2.5 mM ADP and 2.5 μM antimycin A. In between, 0.1 μM H_2_O_2_ was added for internal and experimental calibration. The change in emitted fluorescence, which was directly proportional to the H_2_O_2_ produced, was calculated.

### Statistical analysis

An unpaired two‐tailed Student's *t*‐test was used for comparisons between the two groups. A paired two‐tailed Student's *t*‐test was used for oxygen consumption analysis (*Figure* *7*). A chi‐squared test was used to compare changes in the proportion of nuclear clusters between the control and cachectic muscle tissues. Data are represented as mean ± SEM. Sample size (*n*) refers to biological replicates.

### Data availability

Bulk RNA sequencing data generated in this study are available in the GEO database with Accession Number GSE245799. Single‐nucleus RNA sequencing datasets and analysis codes have been deposited at https://doi.org/10.5281/zenodo.11090497.

## Results

### snRNA‐seq analysis of atrophying muscles identifies mononuclear and myonuclear gene signatures

**Figure 1:**
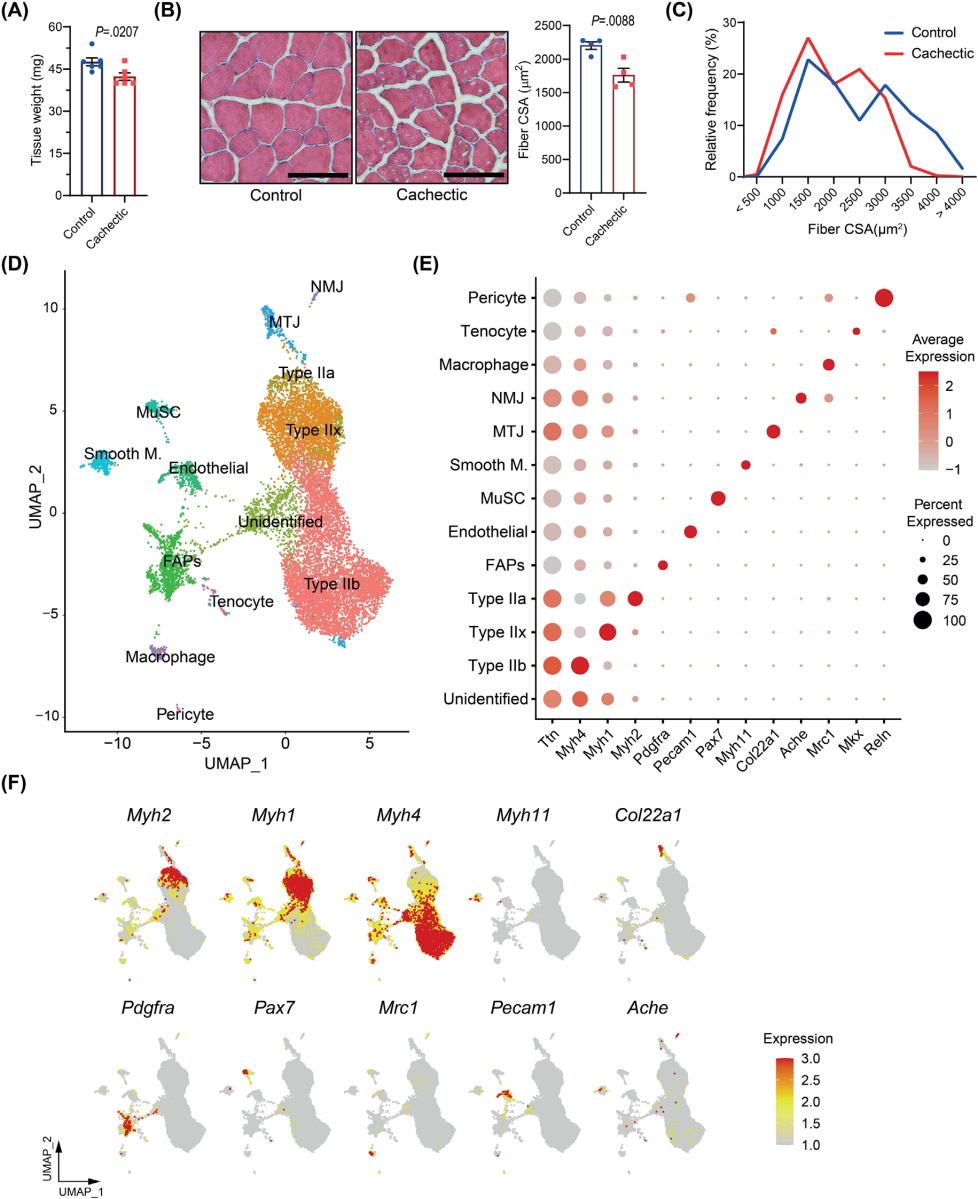
Single‐nucleus RNA sequencing (snRNA‐seq) analysis of atrophying muscles identifies mononuclear and myonuclear gene signatures. (A) Tibialis anterior (TA) muscle weight of control and cachectic mice. (*n* = 6) (B, C) Haematoxylin and eosin staining of the TA muscle cross‐sections. Scale bars: 100 μm. Comparison of mean cross‐sectional areas of TA muscle fibres (*n* = 4) (B) and (C) distribution of fibre frequencies. (D) Uniform manifold approximation and projection (UMAP) visualization of nuclear clusters; each distinct cell type is colour‐coded. (E) Dot plot of marker gene expression for each nuclear cluster. The size of the dots represents the percentage of nuclei expressing the marker gene, and the red colour intensity indicates the expression level. (F) UMAP plots of marker gene expression for each nuclear cluster. (A, B) An unpaired two‐sided Student's *t*‐test was used for statistical analysis. Data are represented as individual points and mean ± SEM. See also *Figure* *S1*.

We utilized LLC cells to induce cachexia and muscle atrophy in mice. Syngeneic C57BL/6 mice injected with LLC cells were sacrificed 16 days after tumour inoculation while they experienced moderate cachexia and loss of muscle mass and function.^5^, ^22^, ^23^, ^24^, ^25^ We investigated single‐nucleus transcriptomes of the TA muscle from tumour‐bearing mice and their non‐tumour‐bearing controls. The TA muscles of the cachectic mice exhibited evident wasting as the tissue weight and average muscle fibre cross‐sectional area were reduced significantly in this group (*Figure* *1* *A,B*). Muscle fibres with a small cross‐sectional area were enriched (*Figure* *1* *C*). Similarly, these mice displayed reduced tumour‐free body weight and wasting of adipose tissues (*Figure* *S1* *A–C*). TA muscle samples from six different mice in each group were pooled for nuclear isolation. DAPI‐stained nuclei were subjected to fluorescence‐activated sorting. We constructed single‐nucleus libraries using 10X Genomics applications, following the manufacturer's guidelines, and performed RNA sequencing. We utilized the Cell Ranger software and the Seurat R package to perform quality control and data analysis, as detailed in the Methods section.

We analysed a total of 12 335 nuclei, comprising 6422 nuclei from the control group and 5892 nuclei from atrophying muscles. Using Seurat and UMAP clustering, we identified 22 distinct unsupervised clusters (*Figure* *S1* *D*). Based on the expression of specific signature genes (*Figure* *S1* *E,F*), all clusters were assigned to 12 known cell types, consisting of type II myofibers (including IIa, IIb and IIx), MTJ, NMJ, FAPs, endothelial cells, MuSCs, smooth muscle cells, macrophages, tenocytes and pericytes, and unidentified nuclei (Cluster 4) (*Figure* *1* *D,E*). A heatmap of the top 5 markers of the identified clusters demonstrated distinct gene expression patterns between these groups (*Figure* *S1* *F*). UMAP clustering of signature genes, including *Myh2* (type IIa), *Myh1* (type IIx), *Myh4* (type IIb), *Myh11* (smooth muscle), *Col22a1* (MTJ), *Pdgfra* (FAPs), *Pax7* (MuSC), *Mrc1* (macrophages), *Pecam1* (endothelial cells) and *Ache* (NMJ), illustrated the distribution of these clusters (*Figure* *1* *F*).

### Tumours induce the enrichment of type IIb myonuclear signatures in tibialis anterior muscle

**Figure 2:**
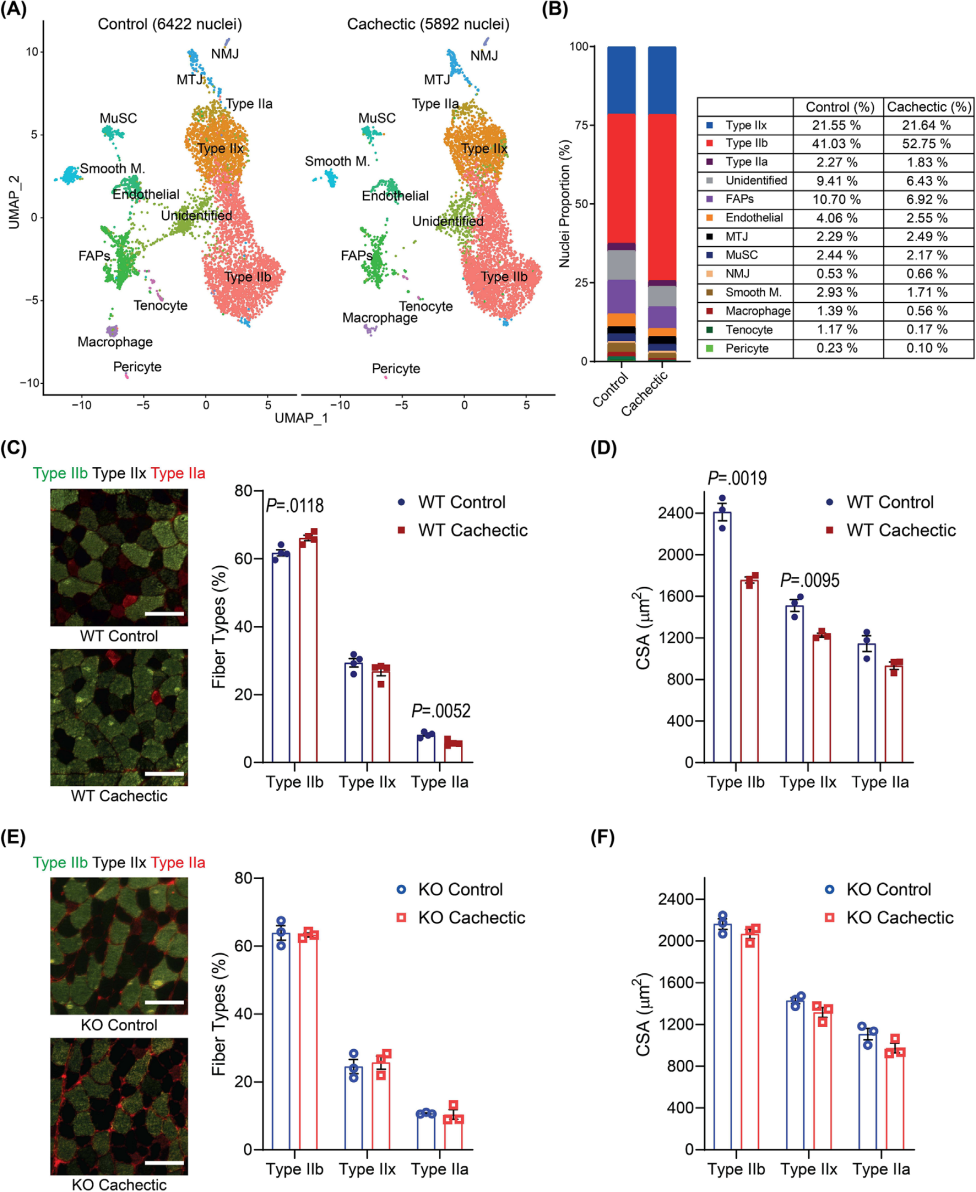
Tumours alter the composition of myofiber types in tibialis anterior (TA) muscle. (A) Uniform manifold approximation and projection (UMAP) plot of nuclear clusters in control (left panel) and cachectic (right panel) TA muscles; each distinct cell type is colour‐coded. (B) Proportions of nuclear clusters in control and cachectic muscles, relative to the total number of nuclei in each group. (C) Representative images of immunofluorescence (IF) staining of TA muscle sections of wild‐type (WT) mice. Green and red signals visualize Myh4 (type IIb) and Myh2 (type IIa) expression, respectively, while dark fibres are type IIx. Scale bars: 100 μm. The fibre‐type composition of WT TA muscle sections identified by IF staining (right panel) (*n* = 4). (D) Comparison of cross‐sectional areas of WT TA muscle fibres (*n* = 3). (E) Representative images of IF staining of TA muscle sections of ectodysplasin A2 receptor (EDA2R) knockout (KO) mice. Scale bars: 100 μm. The fibre‐type composition of the KO TA muscle sections identified by IF staining (right panel) (*n* = 3). (F) Comparison of cross‐sectional areas of KO TA muscle fibres (*n* = 3). (C–F) An unpaired two‐sided Student's *t*‐test was used for statistical analysis. Data are represented as individual points and mean ± SEM.

A comparison of nuclear populations between the control and cachectic muscles indicated that the distribution of these clusters was altered in the presence of tumours (*Figure* *2* *A*). The predominant cell type in the control TA muscles is type II fibres, which contain 64.86% of all nuclei. The representation of type II myonuclei increased to 76.22% in the cachectic muscles (chi‐squared *P* < 0.00001). An increase in the proportion of type IIb myonuclei from 41.03% to 52.75% accounted for the bulk of this change (*Figure* *2* *B*). In addition, slightly increased proportions of NMJ and MTJ myonuclei were detected. Interestingly, the representation of mononuclear cells, including FAPs, endothelial cells, smooth muscle cells, macrophages, tenocytes and pericytes, was reduced in the cachectic muscles (chi‐squared *P* < 0.00001) (*Figure* *2* *B*). These findings argue that tumour growth induced the enrichment of myonuclear signatures, particularly type IIb myonuclei, in muscle tissue.

We analysed the change in the composition of myofibers to investigate the impact of the enrichment of type IIb myonuclear signatures upon tumour inoculation. Immunofluorescence staining of TA muscle sections showed that the majority of muscle fibres are type IIb and type IIx, while type IIa and type I fibres make up a small fraction (*Figure* *2* *C*). The percentage of type IIb fibres increased from 61.59 to 66.11 in the cachectic muscles, arguing that the tumour‐induced increase in the proportion of type IIb myonuclei was also reflected in the frequency of these myofibers (*Figure* *2* *C*). Upon tumour inoculation, the cross‐sectional area of all myofibers was reduced. This effect was more pronounced for type IIb fibres (−27.2%) as opposed to type IIa and type IIx fibres (−18.5% and −18.9%, respectively) (*Figure* *2* *D*). Interestingly, EDA2R‐deficient muscles that are resistant to tumour‐induced muscle loss^5^ did not display the enrichment in type IIb myofibers. Similarly, the cross‐sectional area of the EDA2R‐deficient myofibers did not change significantly upon tumour growth (*Figure* *2* *E*,*F*). Therefore, the switch to type IIb myofiber identity is likely associated with the loss of muscle mass and the activity of the EDA2R pathway.

**Figure 3:**
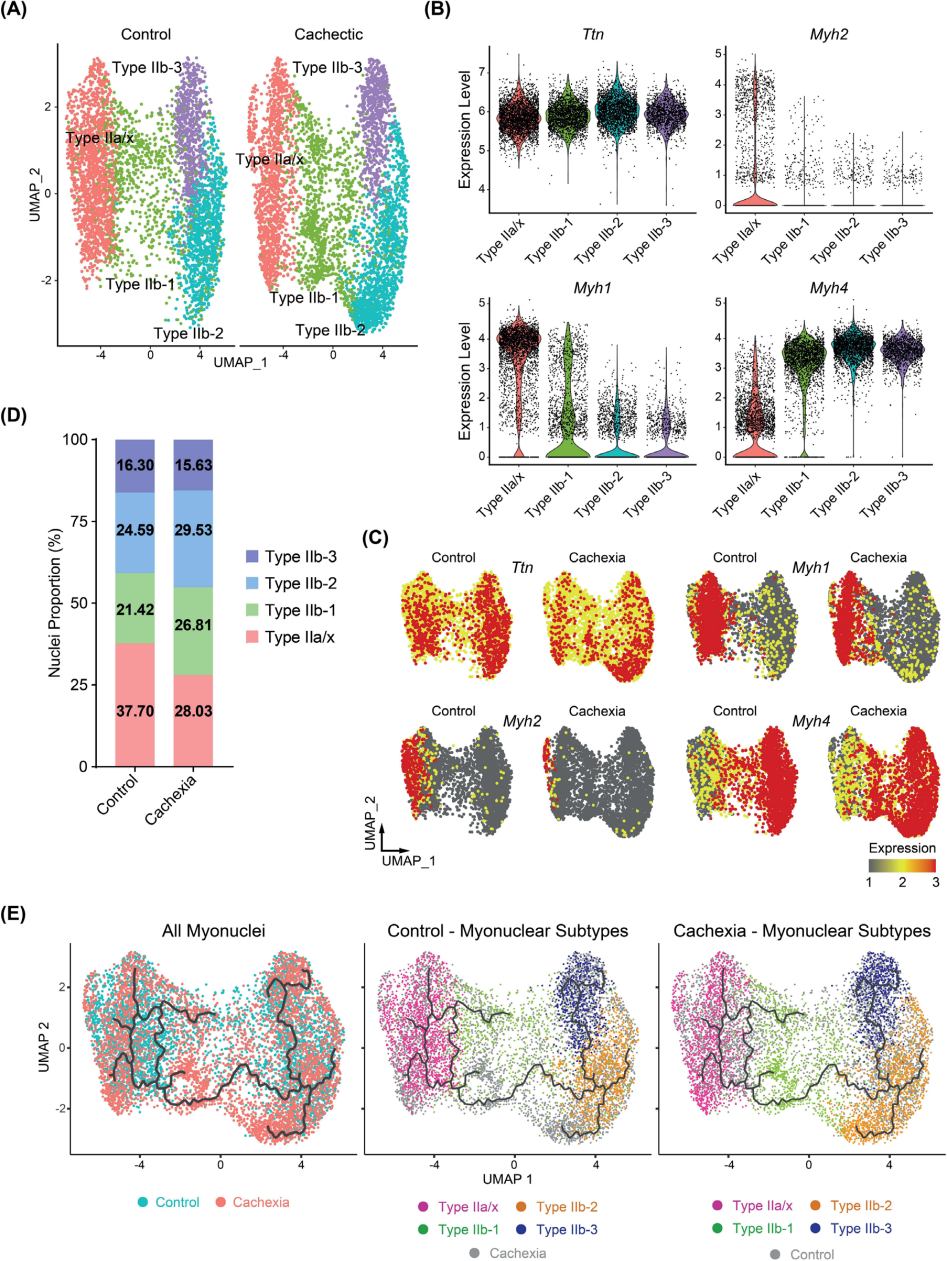
Tumours induce the enrichment of type IIb myonuclear signatures in tibialis anterior (TA) muscle. (A) Uniform manifold approximation and projection (UMAP) plot of myonuclear clusters in control (left panel) and cachectic (right panel) TA muscles; each distinct cell type is colour‐coded. (B) Violin plot of expression levels of the selected genes in myonuclear subtypes. (C) UMAP plots representing levels of marker gene expression in control and cachectic myonuclei. (D) Proportions of myonuclear clusters in control and cachectic muscles. (E) UMAP plots depicting the trajectory analysis of myonuclei from control and cachectic muscles. The myonuclei are coloured according to conditions in the left panel and according to identified subtypes in the middle and right panels. Data are represented as individual points.

We further analysed type II myonuclear gene signatures in our snRNA‐seq dataset and identified four distinct myonuclear populations (*Figure* *3* *A*). We categorized them based on *Myh* gene expression and detected a cluster representing type IIa and type IIx myonuclei (type IIa‐x) and three distinct clusters representing type IIb myonuclei (type IIb‐1, IIb‐2 and IIb‐3) (*Figure* *3* *B,C*). Upon cachexia, a 10% drop in the number of type IIa‐x myonuclei and ~5% increases in each of type IIb‐1 and type IIb‐2 myonuclei were detected (chi‐squared *P* < 0.00001) (*Figure* *3* *D*). In fact, the population of Myh2^+^ myonuclei representing type IIa was clearly diminished with cachexia, whereas Myh4^+^ myonuclei (type IIb) were enriched (*Figure* *3* *C*). Myh1 representation (type IIx) was also reduced. Trajectory analysis of type II myonuclei revealed a continuous path originating from type IIa‐x myonuclei, extending into type IIb‐1 and branching out to type IIb‐2 and type IIb‐3 clusters, implying that cancer cachexia may promote the transition of type IIa and type IIx myonuclei towards the type IIb identity (*Figure* *3* *E*). However, it cannot be ruled out that remote tumour growth also induces the fusion of myogenic progenitors, which contribute myonuclei with type IIb myonuclear characteristics.

### Tumours promote the enrichment of atrophy‐related myonuclear signatures in tibialis anterior muscle

**Figure 4:**
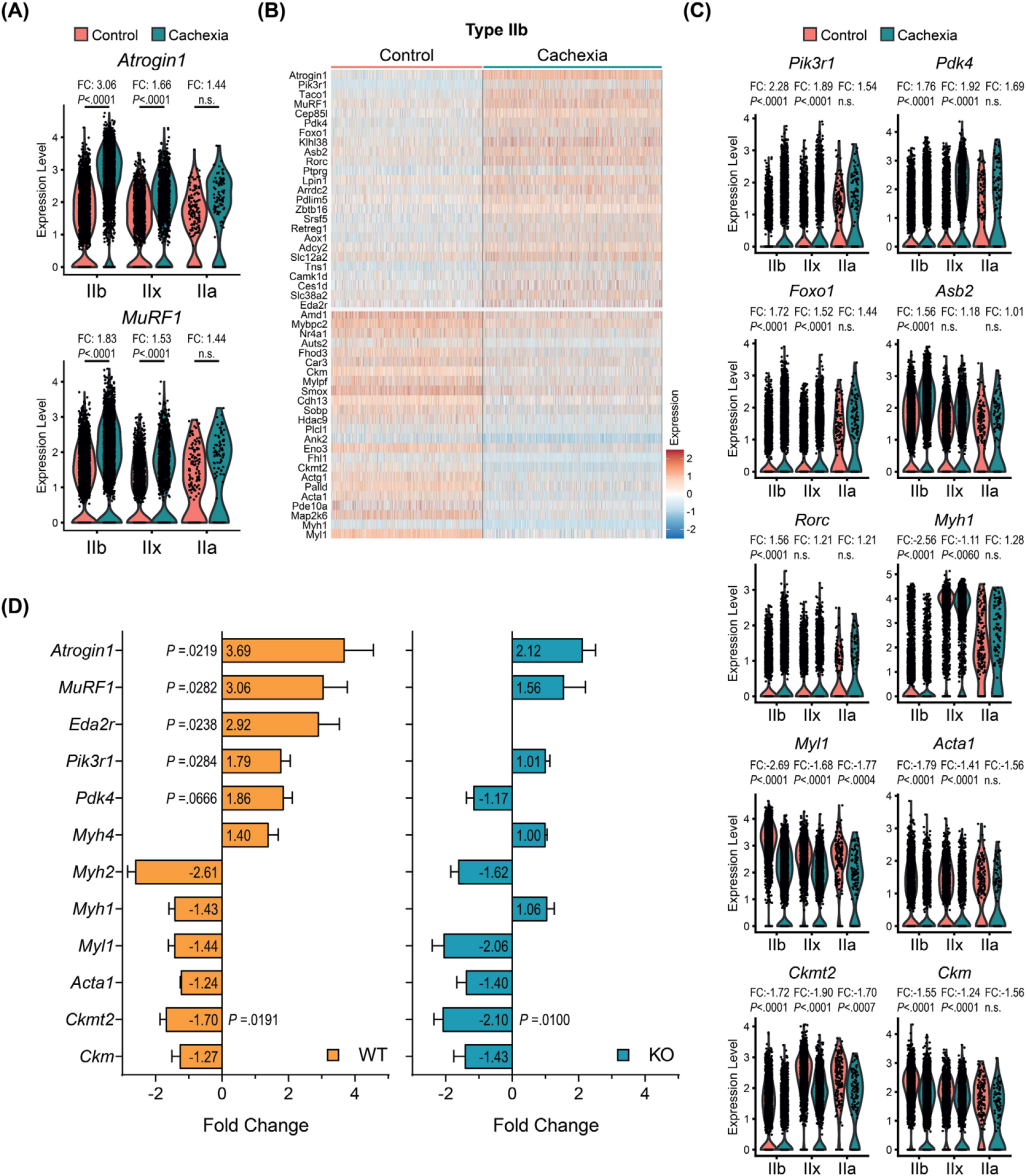
Tumours promote the enrichment of atrophy‐related myonuclear signatures in tibialis anterior (TA) muscle. (A) Violin plots of *Atrogin1* and *MuRF1* expression levels in myonuclei with fold change (FC) values for each cluster (red: control, blue: cachectic). (B) Heatmap of the differentially expressed genes (DEGs) in type IIb myonuclei. (C) Violin plot of the expression levels of selected genes in myonuclei with FC values for each cluster (red: control, blue: cachectic). (D) RT‐qPCR analysis of selected genes in bulk RNA isolated from control and cachectic TA muscle samples of wild‐type (WT) and ectodysplasin A2 receptor (EDA2R) knockout (KO) mice (*n* = 4 for WT, *n* = 5 for KO). (A, C) The Benjamini–Hochberg adjusted *P* value was used for statistical analysis. (D) An unpaired two‐sided Student's *t*‐test was used for statistical analysis. Data are represented as individual points or mean ± SEM.

We next investigated differentially expressed genes (DEGs) in myonuclei. Comparison of control and cachectic muscles indicated upregulated expression of atrophy‐related genes in the latter group, including *Atrogin1* (*Fbxo32*) and *MuRF1* (*Trim63*) (*Figure* *4* *A,B*). These two genes encode E3 ubiquitin ligases, which are well‐known inducers of muscle protein breakdown and atrophy.^4^ The expression of *Atrogin1* and *MuRF1* was elevated in all myofiber types, but this change was particularly striking in type IIb myonuclei (*Figure* *4* *A*), arguing a predominant effect on type IIb fibres during muscle wasting. Other E3 ubiquitin ligase‐associated genes linked to protein degradation, such as *Asb2* and *Klhl38*, were also upregulated (*Figure* *4* *B*).^26^, ^27^ Notably, expression of genes involved in the regulation of metabolic activities, including *Pik3r1*, *Pdk4*, *Foxo1*, *Rorc* and *Lpin1*, increased in type IIb myonuclei upon cachexia (*Figure* *4* *B*). Particularly, *Pdk4*, which encodes a kinase that phosphorylates pyruvate dehydrogenase and inhibits pyruvate oxidation, and the transcription factor *Foxo1* stood out as muscle atrophy‐related markers.^28^, ^29^

Genes downregulated in the cachectic type IIb myonuclei include *Myh1*, *Myl1*, *Acta1*, *Actg1*, *Ank2*, *Fhl1*, *Mylpf*, *Fhod3* and *Mybpc2*, which are involved in muscle contraction and its regulation (*Figure* *4* *B*). Notably, the expression of the creatine kinase genes *Ckm* and *Ckmt2* and the muscle‐specific glycolytic enzyme *Eno3* was also reduced, implicating that the energy metabolism supporting muscle contraction was negatively impacted by tumours (*Figure* *4* *B*). Genes involved in positive regulation of muscle mass, including *Fhl1* and polyamine biosynthesis enzymes *Amd1* and *Smox*, were also downregulated (*Figure* *4* *B*).^30^, ^31^, ^32^ Upregulation or downregulation of the majority of these genes was more pronounced in type IIb myonuclei compared with type IIx and type IIa myonuclei (*Figure* *4* *C*). Type IIb‐enriched gene expression changes were validated by RT‐qPCR analysis of bulk RNA from TA muscle samples (*Figure* *4* *D*). In agreement with the increase in the representation of type IIb myonuclear signatures in cachectic muscle, mRNA levels of Myh1 and Myh2 were reduced, whereas elevated levels of Myh4 were detected. Interestingly, tumour‐induced alterations in the expression of *Atrogin1*, *MuRF1*, *Pik3r1*, *Pdk4*, *Myh4*, *Myh2* and *Myh1* were attenuated in EDA2R‐deficient muscles, implying that the EDA2R pathway contributes to the reprogramming of muscle gene expression during cancer cachexia (*Figure* *4* *D*).

**Figure 5:**
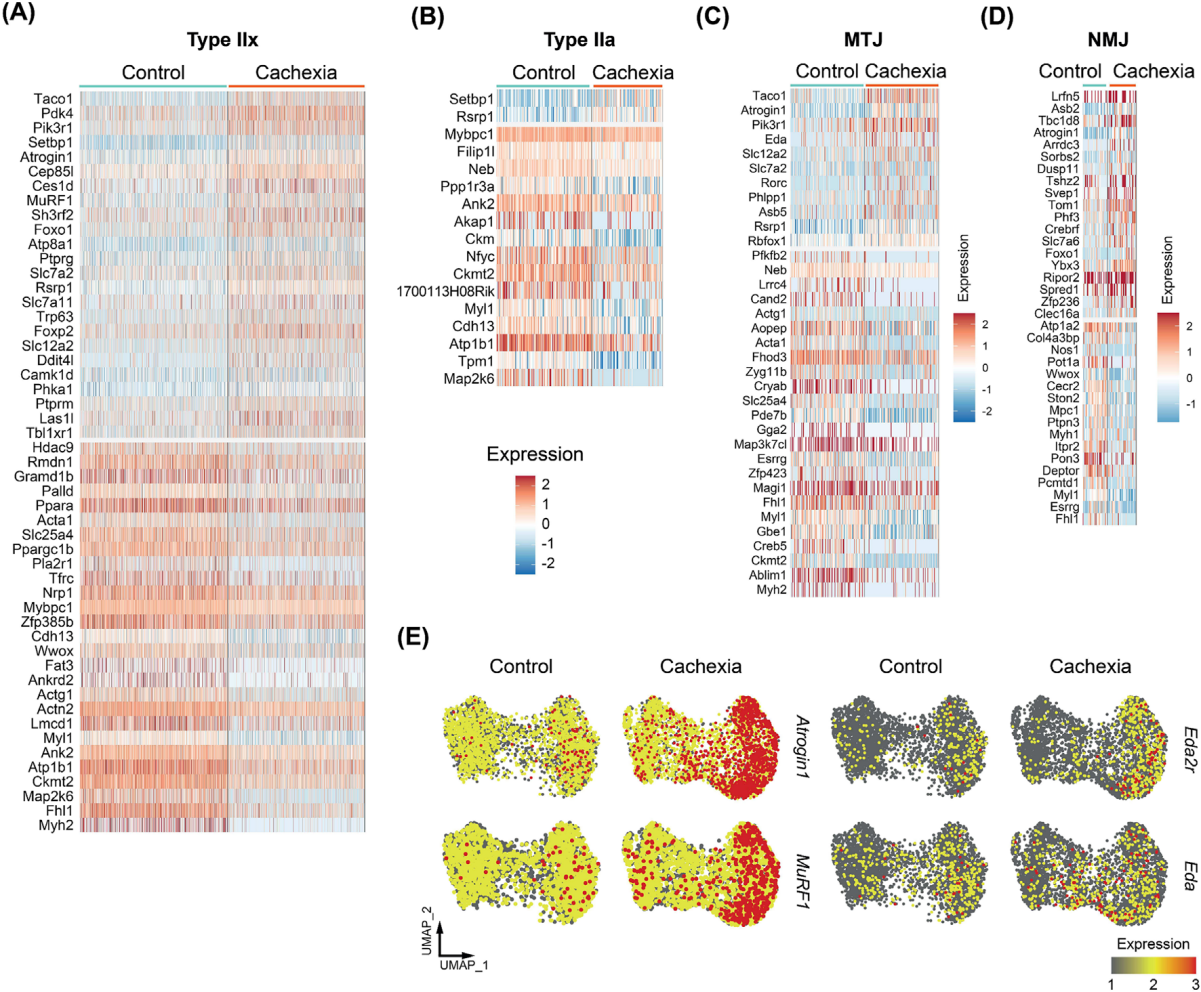
Tumours induce distinct gene expression patterns in myonuclear subclusters of tibialis anterior (TA) muscle. (A–D) Heatmap of the differentially expressed genes (DEGs) in type IIx (A), type IIa (B), myotendinous junction (MTJ) (C) and neuromuscular junction (NMJ) (D) myonuclei. (E) Uniform manifold approximation and projection (UMAP) plots representing expression levels of selected genes in control (left) and cachectic (right) myonuclei.

Distinct gene expression patterns were also detected in type IIx and type IIa myonuclei (*Figure* *5* *A,B*). For instance, transcription factors *Foxp2* and *Ppara* were particularly upregulated and downregulated, respectively, in type IIx myonuclei, while muscle proteins nebulin (*Neb*) and tropomyosin‐1 (*Tpm1*) were suppressed in type IIa myonuclei (*Figure* *5* *A,B*). Upon tumour growth, *Atrogin1* expression was enriched in specialized MTJ and NMJ myonuclei, which also exhibited distinctive changes in their transcriptomes, including increased *Eda* expression in MTJ and reduced *Deptor* and *Nos1* expression in NMJ (*Figure* *5* *C,D*). Additionally, we identified increased expression of *Eda2r*, which showed marked upregulation in the cachectic type IIb myonuclei, a pattern similar to *Atrogin1* and *MuRF1* (*Figures* *4* *B* and *5* *E*). Mild enrichment in *Eda* mRNA is also detectable in type II myonuclei (*Figure* *5* *E*). Our recent work demonstrated that the EDA2R signalling mediates tumour‐induced *Atrogin1* and *MuRF1* expression in muscle tissue, placing EDA2R at upstream of these atrophy markers.^5^

### Ectodysplasin A2 receptor activation and tumour inoculation suppress gene sets related to muscle contraction and oxidative metabolism

**Figure 6:**
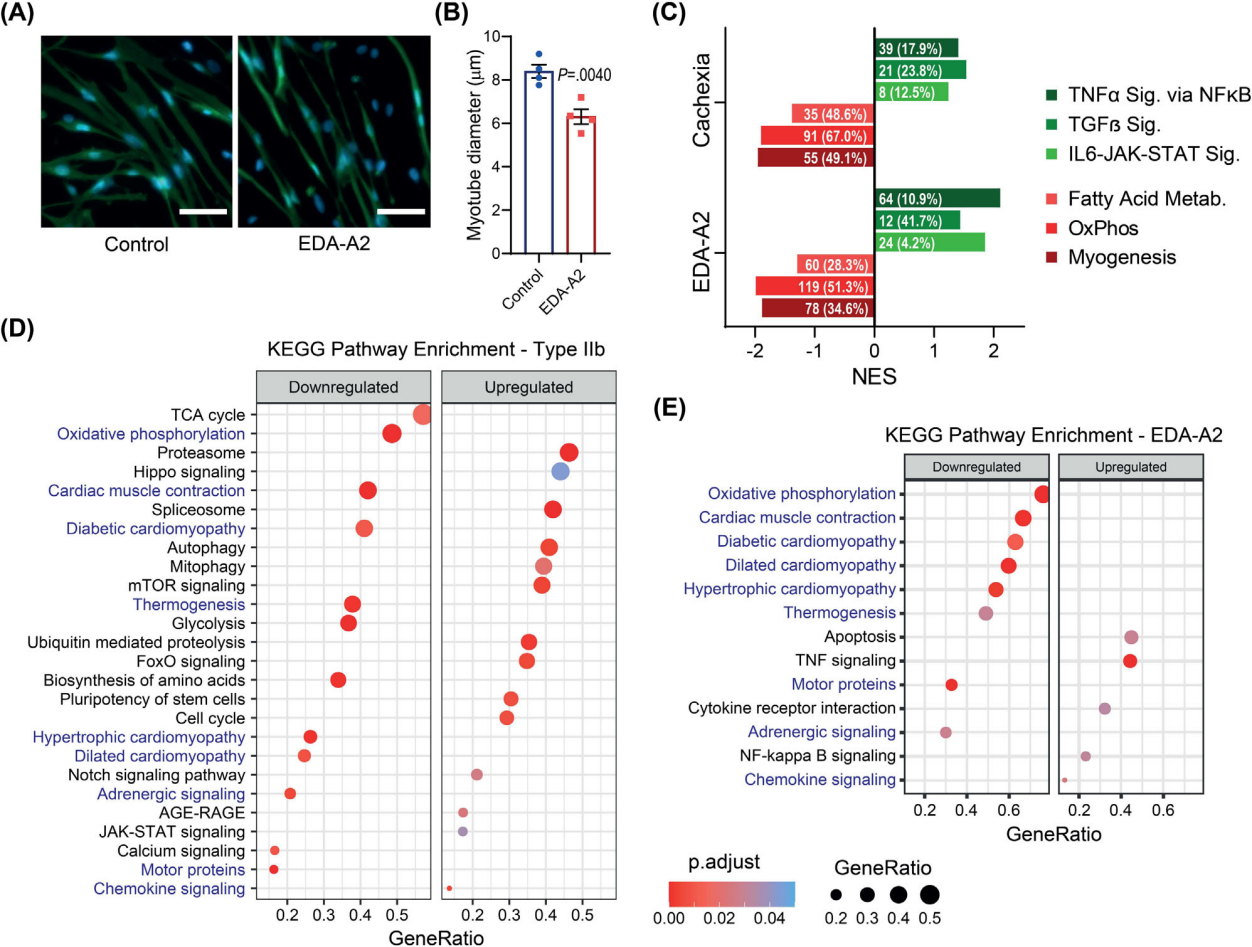
Ectodysplasin A2 receptor (EDA2R) activation and tumour inoculation suppress gene sets related to muscle contraction and oxidative metabolism. (A) Myosin heavy chain immunofluorescence staining of mouse primary myotubes: control (left panel) and recombinant ectodysplasin A2 (EDA‐A2) treatment for 48 h (right panel). Scale bars: 50 μm. (B) Comparison of average myotube diameters between conditions (*n* = 4). (C) Comparison of normalized enrichment score (NES) values of hallmark gene sets enriched in type IIb myonuclei and EDA‐A2‐treated myotubes. Values inside the bars indicate the number of enriched genes and the percentage of common genes between type IIb myonuclei and EDA‐A2‐treated myotubes. (D, E) Kyoto Encyclopedia of Genes and Genomes (KEGG) pathways enriched in cachectic type IIb myonuclei (D) and EDA‐A2‐treated myotubes (E). The size of the dots represents the enriched gene ratio in each pathway, and the red colour intensity represents adjusted *P* values. Blue‐coloured gene sets are common between two groups. (B) An unpaired two‐sided Student's *t*‐test was used for statistical analysis. Data are represented as individual points and mean ± SEM. See also *Figure* *S2* and *Tables* *S1*–*S4*.

Previously, we described muscle atrophy‐inducing effects of the EDA2R pathway.^5^ Stimulation of mouse primary myotubes with EDA‐A2 promoted cellular atrophy detected by myosin heavy chain immunostaining (*Figure* *6* *A*). EDA‐A2 administration significantly reduced myotube diameter (*Figure* *6* *B*). To investigate global transcriptional changes driven by EDA2R activation, we performed bulk RNA sequencing. GSEA of DEGs revealed that hallmark gene sets involving interferon response, inflammatory response, TNFα signalling via nuclear factor‐kappa B (NFκB), interleukin‐6–Janus kinase–signal transducer and activator of transcription (IL6–JAK–STAT) signalling and transforming growth factor‐beta (TGFβ) signalling were enriched in EDA‐A2‐treated myotubes (*Figures* *6* *C* and *S2A* and *Table* *S1*). The roles of these pathways and inflammation in cancer cachexia have been well established.^33^ Gene sets involving myogenesis, oxidative phosphorylation, fatty acid oxidation and angiogenesis were enriched in the control group compared with the EDA‐A2‐treated samples (*Figure* *6* *C* and *Table* *S1*). Because *Eda2r* expression was induced in type IIb myonuclei of cachectic muscles, we performed GSEA of these myonuclei using hallmark gene sets and compared it with the analysis of the EDA‐A2 dataset. Remarkably, pathways activated by EDA‐A2, such as NFκB, JAK–STAT and TGFβ, were also enriched in the cachectic type IIb myonuclei, while pathways suppressed by EDA‐A2, including myogenesis, oxidative phosphorylation and fatty acid oxidation, behaved similarly in the cachectic type IIb myonuclei (*Figures* *6* *C* and *S2B* and *Table* *S2*). Common genes enriched in both datasets are listed in *Table* *S3*. The overlap between the gene set enrichment of EDA‐A2‐treated myotubes and type IIb myonuclei argues that EDA2R upregulation in the cachectic myonuclei likely contributes to the transcriptional reprogramming taking place after tumour inoculation.

We next performed further GSEA analysis of our data using KEGG gene sets. In type IIb myonuclei, gene sets including proteasome, ubiquitin‐mediated proteolysis, autophagy, FOXO signalling, stem cell pluripotency and hippo signalling were upregulated in response to cachexia, while gene sets such as motor proteins, cardiac muscle contraction, cardiomyopathies, glycolysis, oxidative phosphorylation, tricarboxylic acid (TCA) cycle and thermogenesis were downregulated (*Figure* *6* *D*). Remarkably, most gene sets suppressed in cachectic myonuclei were also downregulated in myotubes treated with EDA‐A2, arguing that the EDA2R pathway activation may be related to reduced expression of genes involved in muscle contractility and metabolism (*Figure* *6* *E*). Overlapping genes identified in the enrichment analysis of type IIb myonuclei and EDA‐A2‐treated myotubes are highlighted in *Table* *S3*. Gene set enrichment patterns similar to those of type IIb myonuclei were also detected in type IIx, type IIa, MTJ and NMJ myonuclei (*Figure* *S2C–F*), demonstrating that muscle wasting signatures are not restricted to type IIb myonuclei. However, enrichment of distinct gene sets was also identified in non‐type IIb myonuclei, including Wnt, peroxisome proliferator‐activated receptor (PPAR) and adenosine monophosphate‐activated protein kinase (AMPK) signalling pathways. Notably, the gene set for spliceosome was enriched in all myonuclei upon cachexia, implicating that this process may have an underappreciated role in muscle loss (*Figures* *6* *D* and *S2C–E*). Serine‐ and arginine‐rich splicing factor 5 (*Srsf5*) is particularly upregulated in myonuclei (*Figure* *4* *B*). A complete list of KEGG gene sets regulated in myonuclei is listed in *Table* *S4*.

### Muscle oxidative metabolism is suppressed by tumours and ectodysplasin A2 receptor activation

**Figure 7:**
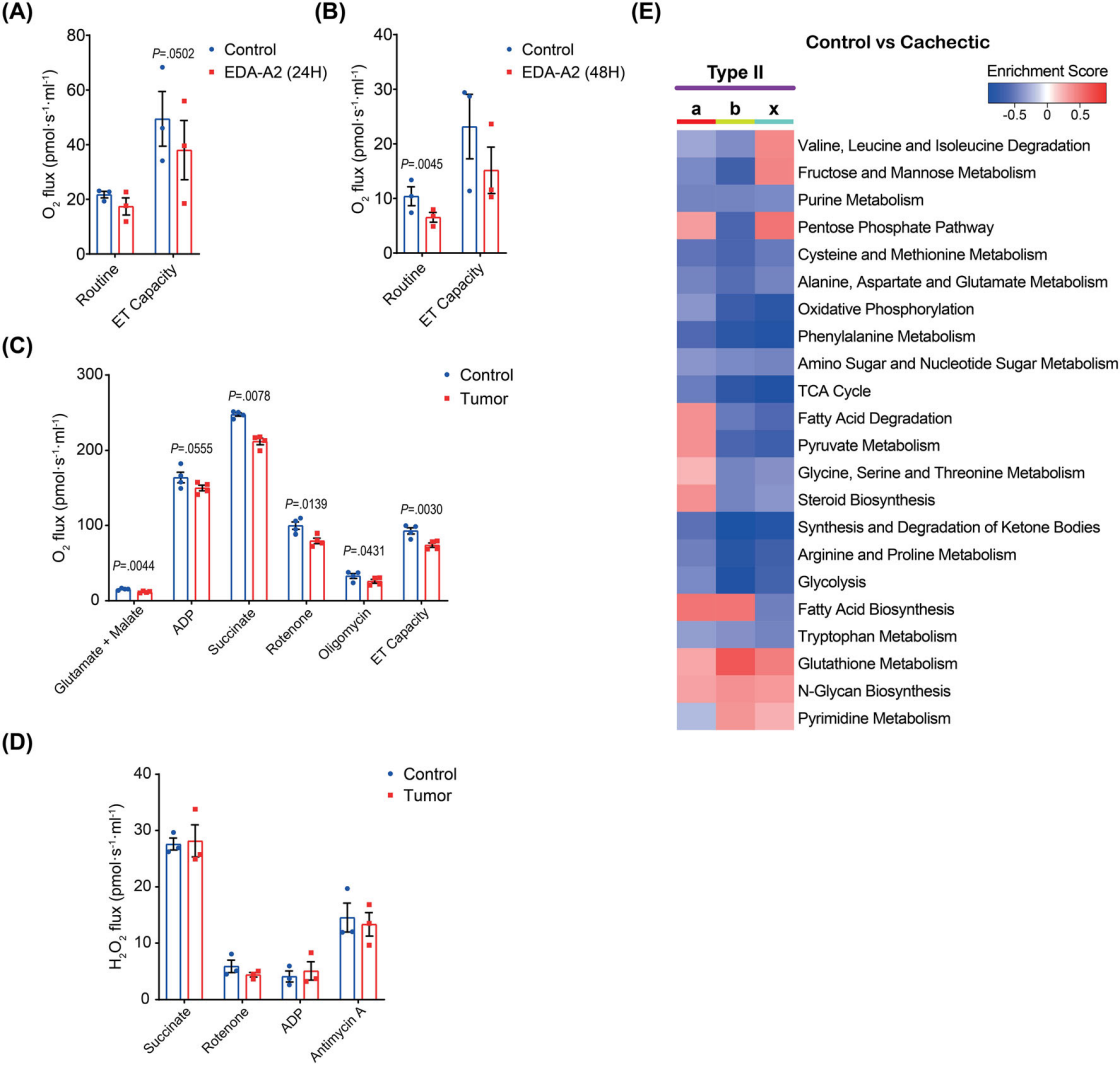
Muscle oxidative metabolism is suppressed by tumours and ectodysplasin A2 (EDA‐A2) receptor (EDA2R) activation. (A, B) O_2_ consumption rates in primary myotubes treated with EDA‐A2 for 24 h (A) and for 48 h (B). Routine represents the basal respiratory activity, and ‘Electron Transfer (ET) capacity’ is the uncoupled respiration obtained by CCCP titration, which represents the maximal O_2_ consumption rate (*n* = 3). (C, D) O_2_ consumption (C) and H_2_O_2_ production (D) rates in mitochondria isolated from tibialis anterior (TA) muscles of control and tumour‐bearing mice (*n* = 4). (E) Heatmap of metabolic pathways enriched in cachectic type II myonuclei. A paired two‐sided Student's *t*‐test was used for statistical analysis. Data are represented as individual points and mean ± SEM.

Intrigued by the suppression of the gene sets for oxidative phosphorylation and thermogenesis in myotubes treated with EDA‐A2 and myonuclei of tumour‐bearing mice, we investigated O_2_ consumption in the muscle cells. Upon measuring basal and maximal O_2_ consumption rates in primary myotubes, we detected a clear trend for reduced oxidative metabolism after the administration of recombinant EDA‐A2 protein (*Figure* *7* *A,B*). We then isolated mitochondria from the TA muscles of control and tumour‐bearing mice and measured their O_2_ consumption rates. Oxygraphic analysis of isolated mitochondria supplemented with both Complex I‐ and Complex II‐linked substrates under phosphorylating, non‐phosphorylating and uncoupled conditions demonstrated decreased mitochondrial respiration in the atrophying muscles (*Figure* *7* *C*). Next, we quantified reactive oxygen species (ROS) generation in skeletal muscle mitochondria by measuring hydrogen peroxide (H_2_O_2_) production. Mitochondrial ROS production was similar in these samples (*Figure* *7* *D*). In agreement with these findings, metabolic pathway enrichment analysis indicated that processes including oxidative phosphorylation, the TCA cycle and glycolysis were downregulated in all type II myonuclei upon cancer cachexia, whereas pyruvate metabolism and fatty acid degradation were suppressed in cachectic type IIb and type IIx myonuclei (*Figure* *7* *E*). Pathways for the metabolism of amino acids, such as alanine, aspartate, glutamate, arginine, proline, valine, isoleucine, isoleucine, tryptophan, phenylalanine, tyrosine, cysteine and methionine, were particularly suppressed in cachectic type IIb myonuclei (*Figure* 7E). In contrast, pathways including N‐glycan biosynthesis and glutathione metabolism were enriched in cachectic type II myonuclei (*Figure* *7* *E*). As type IIb and type IIx myonuclei are more numerous in TA muscles, their metabolic activities are expected to be rather predominant. Overall, our findings indicate that tumour‐induced muscle wasting is associated with reduced oxidative metabolism, including the metabolism of most amino acids in this tissue.

### Tumours alter the transcriptomes of mononuclear cells in muscle tissue

**Figure 8:**
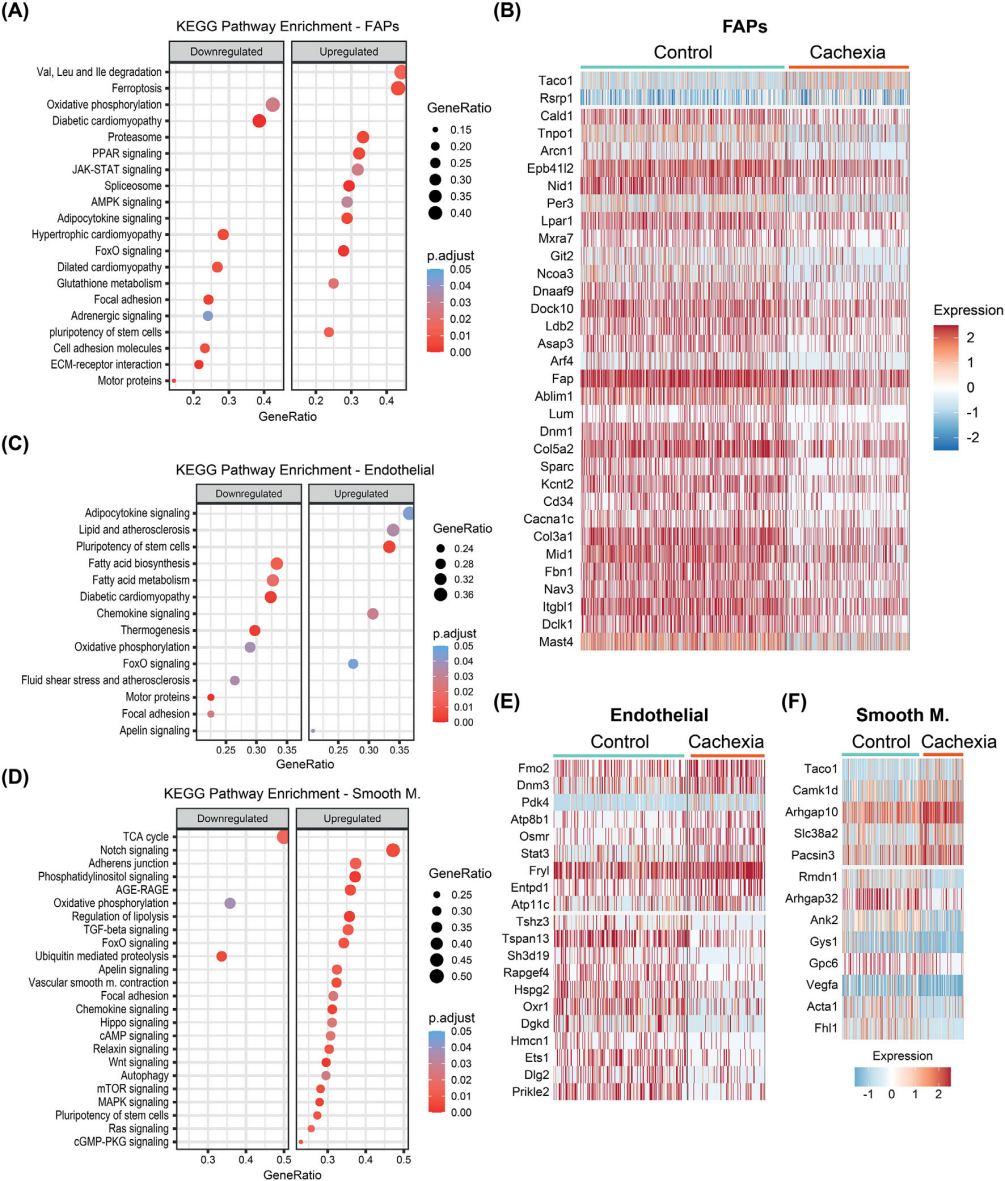
Tumours alter the transcriptomes of mononuclear cells in muscle tissue. (A) Kyoto Encyclopedia of Genes and Genomes (KEGG) pathways enriched in cachectic fibro‐adipogenic progenitor (FAP) nuclei. (B) Heatmap of the differentially expressed genes (DEGs) in FAP nuclei. (C, D) KEGG pathways enriched in cachectic endothelial nuclei (C) and cachectic smooth muscle nuclei (D). The size of the dots represents the enriched gene ratio in each pathway, and the red colour intensity represents adjusted *P* values. (E, F) Heatmap of the DEGs in endothelial nuclei (E) and smooth muscle nuclei (F).

We also investigated differential gene expression in the nuclei of mononuclear cells in skeletal muscle. The most numerous mononuclear cells—FAPs, endothelial and smooth muscle cells—exhibited striking expression patterns. FAPs gene signatures involved tumour‐induced suppression of gene sets associated with oxidative phosphorylation and cardiomyopathy and the enrichment of gene sets associated with the proteasome, spliceosome, JAK–STAT signalling and FOXO signalling, which are reminiscent of myonuclear transcriptional changes. In addition, FAPs nuclei also displayed the upregulation of gene sets for amino acid degradation, ferroptosis and adipocytokine signalling and the downregulation of gene sets for focal adhesion and extracellular matrix (ECM)–receptor interaction (*Figure* *8* *A*). Notably, the heatmap of transcripts with the highest expression changes in FAPs was populated by genes with ECM‐related functions, including *Nid1*, *Fap*, *Lum*, *Sparc*, *Cd34*, *Fbn1*, *Itgbl1* and *Mxra7*, which were suppressed in the cachectic muscles (*Figure* *8* *B*).

Gene expression analysis in endothelial and smooth muscle cells of muscle vasculature also revealed the suppression of gene sets associated with oxidative metabolism in response to tumours (*Figure* *8* *C,D*). Therefore, it is likely that reduced O_2_ consumption is relevant for both myofibers and the majority of mononuclear cells in the cachectic muscle. Upon tumour growth, genes involved in angiogenesis and vascular growth, such as *Vegfa* and *Fhl1*, were suppressed in smooth muscle cells, while *Hspg2* (perlecan) and *Ets1* were downregulated in endothelial cells (*Figure* *8* *E,F*).^34^, ^35^, ^36^ In addition, muscle atrophy‐related genes *Osmr* and *Stat3* were upregulated in endothelial cells (*Figure* *8* *F*).^25^ Notably, KEGG gene sets for lipid and pro‐atherosclerosis and adipocytokine signalling were enriched in the endothelial cells of the cachectic muscles, whereas fluid shear stress and atherosclerosis (mostly anti‐atherosclerotic factors) and focal adhesion gene sets were suppressed (*Figure* *8* *C*). Gene signatures in smooth muscle cells in the cachectic muscles exhibited the enrichment of gene sets, including Notch signalling, adherens junction, focal adhesion, vascular smooth muscle contraction, cyclic guanosine monophosphate‐protein kinase G (cGMP‐PKG) signalling and autophagy, implying that these cells undergo significant remodelling under cachectic conditions (*Figure* *8* *D*).

## Discussion

The syncytial nature of myofibers is a major obstacle to a high‐resolution analysis of gene expression in skeletal muscle. scRNA‐seq is not suitable for studying myonuclear transcriptomes because this technique detects very few muscle cells.^37^ However, snRNA‐seq is a reliable alternative that identifies both myonuclear and mononuclear transcriptomes in muscle tissue.^9^, ^10^ It should be noted that snRNA‐seq only detects nuclear‐enriched transcripts, including pre‐mRNA, and therefore, alterations in the cytoplasmic mRNA pool remain hidden. As cytoplasmic transcripts are unaccounted for, the resolution is not optimal for genes with low expression levels. Despite these limitations, snRNA‐seq analysis of muscle tissue has been used to document the impact of disease states, such as Duchenne muscular dystrophy and denervation, on transcript levels.^12^, ^14^, ^16^ In this study, we utilized snRNA‐seq to study the cachectic skeletal muscle of tumour‐bearing mice. Our results revealed that remote tumour growth transforms gene expression in mononuclear cells and myonuclei. Analysis of DEGs indicated the adoption of myonuclear gene signatures associated with muscle atrophy‐related processes, such as enhanced protein degradation and reduced oxidative metabolism.

snRNA‐seq analysis enables the determination of myofiber‐type‐specific gene signatures in myonuclei. We demonstrated that type IIb myonuclei were enriched in the cachectic muscles. From these data, it is unclear if cachexia induced the emergence of new type IIb myonuclei or the adoption of a type IIb‐resembling signature in the existing myonuclei via transcriptional reprogramming. However, pseudotime trajectory analysis indicated that a transition from type IIa‐x myonuclei towards the type IIb identity is possible. Interestingly, the increase in the proportion of type IIb myonuclei is also reflected in the frequency of type IIb myofibers. In agreement with previous studies that reported a transition towards type IIb myofibers in cachectic muscles,^38^, ^39^, ^40^ a slight increase in the percentage of these myofibers was also detected in the TA muscle. Whether tumour‐induced enrichment of type IIb myonuclear signatures is limited to TA muscle remains to be determined. This muscle tissue is enriched in type II fibres, while it is a scarce source of type I fibres. Therefore, the current study was unable to determine how tumour‐induced cachexia alters gene expression signatures in type I fibres. Additional studies should address how cancer cachexia changes myonuclear identity and gene expression profiles in different muscle tissues, including the soleus muscle, which contains a significant fraction of type I fibres.

Profound upregulation of atrophy‐related genes, including E3 ubiquitin ligases *Atrogin1* and *MuRF1*, transcription factor *Foxo1* and pyruvate dehydrogenase kinase *Pdk4*, was detected in myonuclei isolated from the cachectic muscles.^28^, ^29^ While *Asb2* as a negative regulator of muscle mass was induced in these samples, positive regulators of muscle mass, such as *Fhl1*, *Amd1* and *Smox*, were downregulated.^26^, ^30^, ^31^, ^32^ This was accompanied by reduced levels of genes involved in muscle contraction and energy metabolism, including *Myh1*, *Myl1*, *Acta1*, *Eno3*, *Ckm* and *Ckmt2*. Notably, differential gene expression related to muscle atrophy and metabolism was more pronounced in type IIb myonuclei, in which *Eda2r* is also upregulated. EDA2R signalling has recently been described as playing a prominent role in tumour‐induced muscle wasting.^5^ It is likely that EDA2R upregulation in the cachectic myonuclei contributes to the transcriptional reprogramming taking place after tumour inoculation. In fact, GSEA demonstrated that EDA2R activation and tumour inoculation led to similar expression patterns in muscle cells, including the stimulation of NFκB, JAK–STAT and TGFβ pathways and the suppression of myogenesis, oxidative phosphorylation and fatty acid oxidation. Our flux measurements indicated reduced O_2_ consumption in myotubes upon the activation of EDA2R signalling. In agreement with a previous study,^41^ mitochondrial respiration was also suppressed in cachectic muscles. Reduced mitochondrial oxidation and inefficient metabolism could potentially support muscle wasting. However, elevated energy expenditure and O_2_ consumption were previously described in tumour‐bearing mice, which were attributed to enhanced adipose tissue browning.^22^, ^23^ Our findings argue that skeletal muscle metabolism with reduced oxidative potential is unlikely to contribute directly to the accelerated metabolic rate prevalent in cachectic mice.

snRNA‐seq analysis of mononuclear cells in the cachectic muscles also revealed substantial transcriptional changes, including the downregulation of genes with ECM‐related functions in FAPs and the downregulation of genes involved in vascular growth in smooth muscle and endothelial cells. The suppression of gene sets associated with oxidative metabolism in these cells argues that impairment in mitochondrial respiration is not restricted to myofibers, and this effect may stem from a deficiency in the vasculature of the cachectic muscle tissue. In fact, a recent study utilizing cachexia‐inducing tumour models described weakened vascular functions in atrophying muscles.^42^ In skeletal muscle tissue, tumours likely exert an extensive impact that does not merely target myofibers. Finally, the gene sets identified in this study contribute significantly to our understanding of potential therapeutic targets for mitigating cancer cachexia and associated muscle wasting. Future research should focus on uncovering the mechanisms involving these genes and their specific roles in muscle wasting.

## Conflict of interest statement

The authors declare no conflict of interest.

## Funding information

This work was supported by the EMBO Installation Grant (#4162) and the Scientific and Technological Research Council of Turkey (TÜBİTAK) Grant 122Z163 to S. Kir.

## Supporting information


**Figure S1** Single‐nucleus RNA‐seq analysis of atrophying muscles identifies distinct nuclear signatures. Related to Figure 1. (A) Tumor‐free body weight of control and cachectic mice (*n* = 6). (B) Tumor weight of cachectic mice (n = 6). (C) Epididymal white adipose tissue (Epi), inguinal white adipose tissue (Ing), and interscapular brown adipose tissue (Bat) weight of control and cachectic mice (*n* = 6). (D) UMAP plot of color‐coded unsupervised clusters. (E) Dot plot of marker gene expression in unsupervised clusters. The size of the dots represents the percentage of nuclei expressing the marker gene and the red color intensity indicates the expression level. (F) Heatmap of the top 5 signature genes of each nuclear cluster. (A,C) Unpaired two‐sided Student's t‐test was used for statistical analysis. Data are represented as individual points and mean ± SEM.
**Figure S2.** EDA2R activation and tumor inoculation suppress gene sets related to muscle contraction and oxidative metabolism. Related to Figure 6. (A,B) GSEA plots of hallmark gene sets enriched in EDA‐A2‐treated myotubes (A) and cachectic type IIb myonuclei (B). NES, normalized enrichment score; FDR, false discovery rate. (C‐F) KEGG pathways enriched in cachectic type IIx (C), type IIa (D), MTJ (E), and NMJ (F) myonuclei. The size of the dots represents the enriched gene ratio in each pathway and the red color intensity represents adjusted p values. Blue colored gene sets are shared with the cachectic type IIb myonuclei.

**Table S1.** Differential Expression Analysis of Hallmark Gene Sets in EDA‐A2 treated myotubes. Related to Figure 6.

**Table S2.** Gene Set Enrichment Analysis in Type IIb myonuclei: Cachectic vs Control. Related to Figure 6.

**Table S3.** Gene Sets and Genes Enriched in both EDA‐A2‐treated Myotubes and Cachectic Type IIb Myonuclei. Related to Figure 6.

**Table S4.** nRNA‐Seq KEGG Pathway Analysis. Related to Figure 6 and S2.
