# Supplementary material for: Tumour‐induced alterations in single‐nucleus transcriptome of atrophying muscles indicate enhanced protein degradation and reduced oxidative metabolism
Source: J Cachexia Sarcopenia Muscle. 2024 Jul 13;15(5):1898–914. doi: 10.1002/jcsm.13540 (PMC11446705; doi:10.1002/jcsm.13540)
Supplement: Supplementary file 1 — Figure S1 Single‐nucleus RNA‐seq analysis of atrophying muscles identifies distinct nuclear signatures. Related to Figure 1. (A) Tumor‐free body weight of control and cachectic mice (n = 6). (B) Tumor weight of cachectic mice (n = 6). (C) Epididymal white adipose tissue (Epi), inguinal white adipose tissue (Ing), and interscapular brown adipose tissue (Bat) weight of control and cachectic mice (n = 6). (D) UMAP plot of color‐coded unsupervised clusters. (E) Dot plot of marker gene expression in unsupervised clusters. The size of the dots represents the percentage of nuclei expressing the marker gene and the red color intensity indicates the expression level. (F) Heatmap of the top 5 signature genes of each nuclear cluster. (A,C) Unpaired two‐sided Student's t‐test was used for statistical analysis. Data are represented as individual points and mean ± SEM. Figure S2. EDA2R activation and tumor inoculation suppress gene sets related to muscle contraction and oxidative metabolism. Related to Figure 6. (A,B) GSEA plots of hallmark gene sets enriched in EDA‐A2‐treated myotubes (A) and cachectic type IIb myonuclei (B). NES, normalized enrichment score; FDR, false discovery rate. (C‐F) KEGG pathways enriched in cachectic type IIx (C), type IIa (D), MTJ (E), and NMJ (F) myonuclei. The size of the dots represents the enriched gene ratio in each pathway and the red color intensity represents adjusted p values. Blue colored gene sets are shared with the cachectic type IIb myonuclei. [file JCSM-15-1898-s001.pdf]

Tumor-induced alterations in single-nucleus transcriptome of atrophying muscles indicate enhanced protein degradation and reduced oxidative metabolism

Samet Agca[1], Aylin Domaniku-Waraich[1], Sevval Nur Bilgic[1], Melis Sucuoglu[1], Meric Dag[2], Sukru Anil Dogan[2], Serkan Kir[*,1]

1. Department of Molecular Biology and Genetics, Koc University, Istanbul 34450, Turkey

2. Department of Molecular Biology and Genetics, Center for Life Sciences and Technologies, Bogazici University, Istanbul 34342, Turkey

*Correspondence: skir@ku.edu.tr.

Supplemental information

Fig S1 Single-nucleus RNA-seq analysis of atrophying muscles identifies distinct nuclear signatures. Related to Fig 1. (A) Tumor-free body weight of control and cachectic mice (n=6). (B) Tumor weight of cachectic mice (n=6). (C) Epididymal white adipose tissue (Epi), inguinal white adipose tissue (Ing), and interscapular brown adipose tissue (Bat) weight of control and cachectic mice (n=6). (D) UMAP plot of color-coded unsupervised clusters. (E) Dot plot of marker gene expression in unsupervised clusters. The size of the dots represents the percentage of nuclei expressing the marker gene and the red color intensity indicates the expression level. (F) Heatmap of the top 5 signature genes of each nuclear cluster. (A,C) Unpaired two-sided Student's t-test was used for statistical analysis. Data are represented as individual points and mean ± SEM.

Fig S2 EDA2R activation and tumor inoculation suppress gene sets related to muscle contraction and oxidative metabolism. Related to Fig 6. (A,B) GSEA plots of hallmark gene sets enriched in EDA-A2-treated myotubes (A) and cachectic type IIb myonuclei (B). NES, normalized enrichment score; FDR, false discovery rate. (C-F) KEGG pathways enriched in cachectic type IIx (C), type IIa (D), MTJ (E), and NMJ (F) myonuclei. The size of the dots represents the enriched gene ratio in each pathway and the red color intensity represents adjusted p values. Blue colored gene sets are shared with the cachectic type IIb myonuclei.

Table S1 Differential Expression Analysis of Hallmark Gene Sets in EDA-A2 treated myotubes. Related to Fig 6.

The results of the gene set enrichment analysis comparing EDA-A2-treated myotubes with control samples are presented. The analysis involves the hallmark gene sets.

NAME: Name of the hallmark gene set, SIZE: Number of genes in the gene set, ES (Enrichment Score): The score reflecting the degree to which the gene set is overrepresented at the top or bottom of the ranked list of genes, NES (Normalized Enrichment Score): The ES normalized for differences in gene set size, NOM p-val (Nominal p-Value): The statistical significance of the ES, FDR q-val (False Discovery Rate q-Value): Adjusted p-value accounting for multiple hypothesis testing, FWER p-val (Family-Wise Error Rate p-Value): The probability of at least one Type I error among all the hypothesis tests, RANK AT MAX: The position in the ranked list of genes at which the ES is at its maximum.

Table S2 Gene Set Enrichment Analysis in Type IIb myonuclei: Cachectic vs Control. Related to Fig 6.

This table details the gene set enrichment analysis using the Hallmark gene sets and comparing cachectic and control type IIb myonuclei. The definition of abbreviations is the same as Table S1.

Table S3 Gene Sets and Genes Enriched in both EDA-A2-treated Myotubes and Cachectic Type IIb Myonuclei. Related to Fig 6.

This table lists gene sets and genes enriched in both EDA-A2 treated myotubes and cachectic type IIb myonuclei. This table contains two sheets; one for the Hallmark gene sets and another for the KEGG gene sets.

Sample: Name of the sample, Gene Set: Name of the common enriched gene set, Set Size: Number of genes in the gene set, ES (Enrichment Score): The score reflecting the degree to which the gene set is overrepresented at the top or bottom of the ranked list of genes, NES (Normalized Enrichment Score): The ES normalized for differences in gene set size, qvalue (False Discovery Rate q-Value): Adjusted p-value

accounting for multiple hypothesis testing, Core Enrichment: List of core enriched genes in the pathway, common genes colored with green.

Table S4 snRNA-Seq KEGG Pathway Analysis. Related to Fig 6 and S2.

This table series includes the results of the gene set enrichment analysis of the single-nucleus RNA sequencing data. Each sheet in this table represents a distinct nuclear cluster, and the data is analyzed with respect to the KEGG pathways. ID: KEGG pathway identifier, Description: Description of the KEGG pathway, setSize: Number of genes in the pathway, Enrichment Score: Quantifies the degree to which a set of genes is overrepresented at the top or bottom of a ranked list of genes, NES (Normalized Enrichment Score): Adjusts the Enrichment Score for the size of the gene set, P-value: Significance of the enrichment score, Q-value: False discovery rate adjusted p-value, Rank: Rank of the gene set in the analysis, Leading Edge: Percentage representation of the gene set in the analysis, Core Enrichment: List of core enriched genes in the pathway.

Figure S1

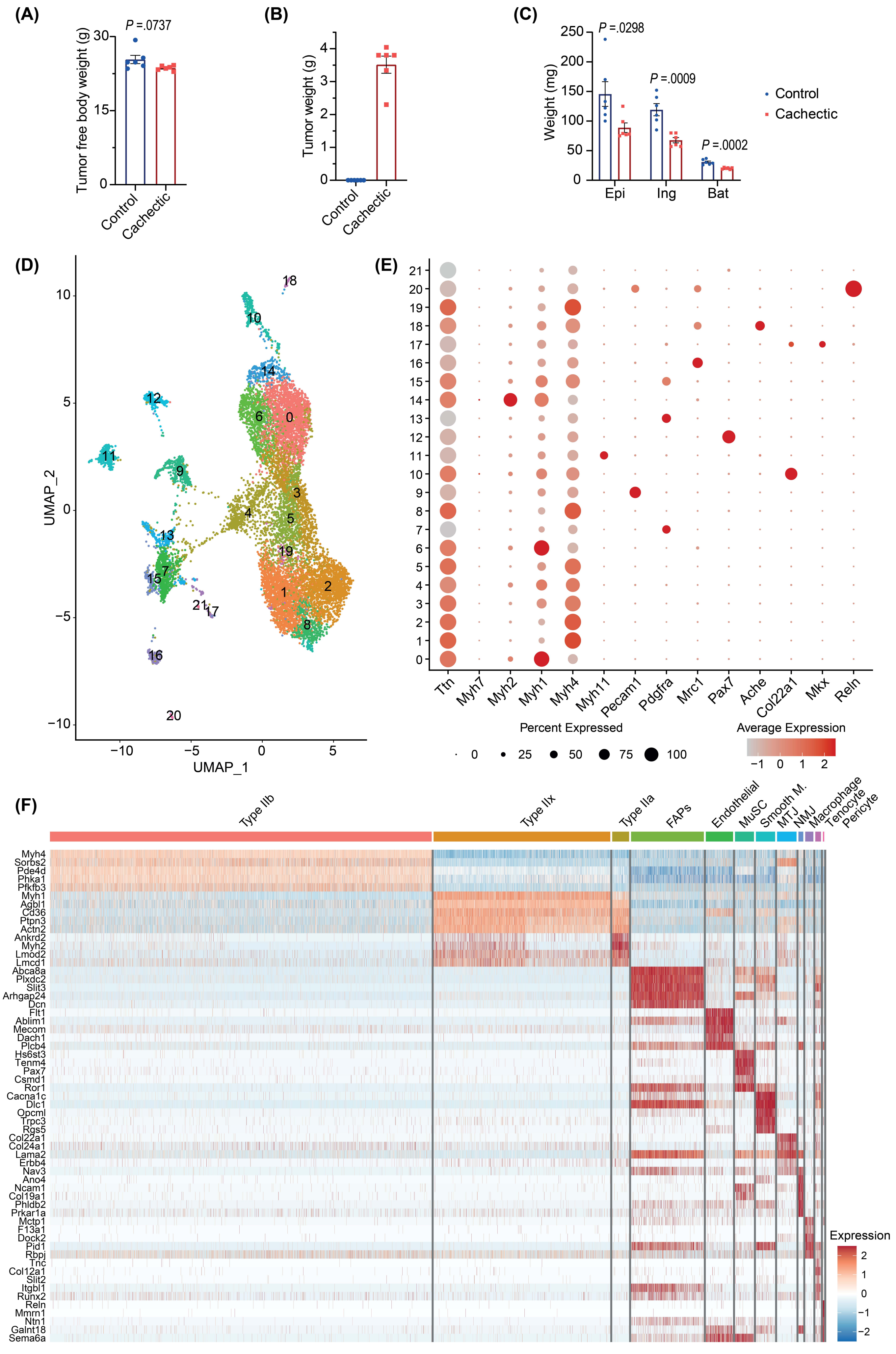
(A)
P =.0737
Tumor free body weight (g)
30
15
0
Control
Cachectic
(B)
Tumor weight (g)
4
3
2
1
0
Control
Cachectic
(C)
P =.0298
P =.0009
P =.0002
Weight (mg)
250
200
150
100
50
0
Epi
Ing
Bat
Control
Cachectic
(D)
UMAP_2
UMAP_1
(E)
Ttn
Myh7
Myh2
Myh1
Myh4
Myh11
Pecam1
Pdgfra
Mrc1
Pax7
Ache
Col22a1
Mkx
Reln
Percent Expressed
0
25
50
75
100
Average Expression
(F)
Type IIb
Type IIx
Type IIa
FAPs
Endothelial
MuSC
Smooth M.
MTJ
NMJ
Macrophage
Tenocyte
Pericyte
Myh4
Sorbs2
Pde4d
Phka1
Pfkfb3
Myh1
Agbl1
Cd36
Ptpn3
Actn2
Ankrd2
Myh2
Lmod2
Lmcd1
Abca8a
Plxdc2
Slit3
Arhgap24
Dcn
Flt1
Ablim1
Mecom
Dach1
Plcb4
Hs6st3
Tenm4
Pax7
Csmd1
Ror1
Cacna1c
Dlc1
Opcml
Trpc3
Rgs5
Col22a1
Col24a1
Lama2
Erbb4
Nav3
Ano4
Ncam1
Col19a1
Phldb2
Prkar1a
Mctp1
F13a1
Dock2
Pid1
Rbpj
Tnc
Col12a1
Slit2
Itgbl1
Runx2
Reln
Mmrn1
Ntn1
Galnt18
Sema6a
Expression

Figure S2

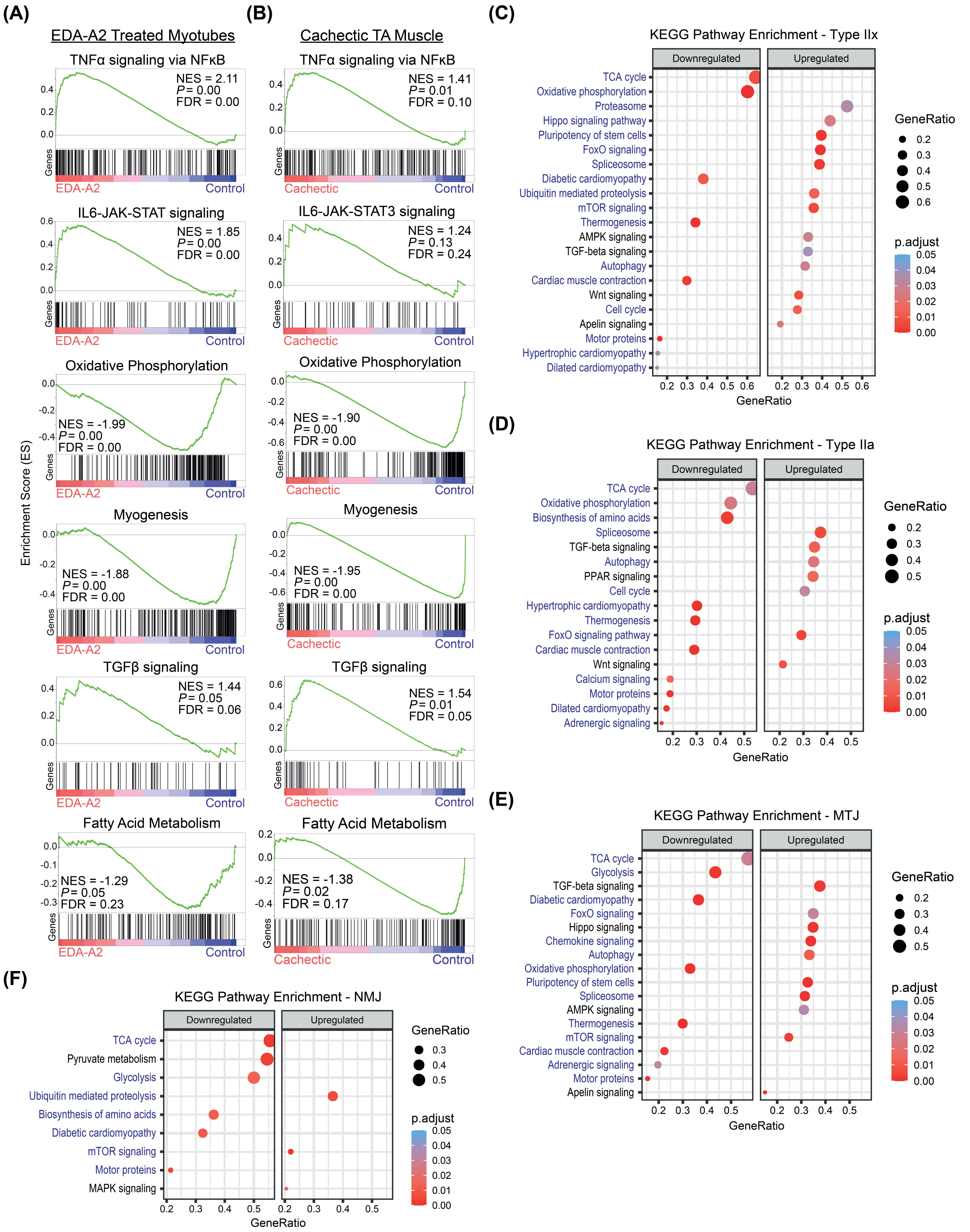

(A)
EDA-A2 Treated Myotubes
TNFα signaling via NFκB
NES = 2.11
P = 0.00
FDR = 0.00
IL6-JAK-STAT signaling
NES = 1.85
P = 0.00
FDR = 0.00
Oxidative Phosphorylation
NES = -1.99
P = 0.00
FDR = 0.00
Myogenesis
NES = -1.88
P = 0.00
FDR = 0.00
TGFβ signaling
NES = 1.44
P = 0.05
FDR = 0.06
Fatty Acid Metabolism
NES = -1.29
P = 0.05
FDR = 0.23
EDA-A2
Control
Genes
Enrichment Score (ES)
(B)
Cachectic TA Muscle
TNFα signaling via NFκB
NES = 1.41
P = 0.01
FDR = 0.10
IL6-JAK-STAT3 signaling
NES = 1.24
P = 0.13
FDR = 0.24
Oxidative Phosphorylation
NES = -1.90
P = 0.00
FDR = 0.00
Myogenesis
NES = -1.95
P = 0.00
FDR = 0.00
TGFβ signaling
NES = 1.54
P = 0.01
FDR = 0.05
Fatty Acid Metabolism
NES = -1.38
P = 0.02
FDR = 0.17
Cachectic
Control
(C)
KEGG Pathway Enrichment - Type IIx
Downregulated
Upregulated
TCA cycle
Oxidative phosphorylation
Proteasome
Hippo signaling pathway
Pluripotency of stem cells
FoxO signaling
Spliceosome
Diabetic cardiomyopathy
Ubiquitin mediated proteolysis
mTOR signaling
Thermogenesis
AMPK signaling
TGF-beta signaling
Autophagy
Cardiac muscle contraction
Wnt signaling
Cell cycle
Apelin signaling
Motor proteins
Hypertrophic cardiomyopathy
Dilated cardiomyopathy
GeneRatio
p.adjust
(D)
KEGG Pathway Enrichment - Type IIa
Downregulated
Upregulated
TCA cycle
Oxidative phosphorylation
Biosynthesis of amino acids
Spliceosome
TGF-beta signaling
Autophagy
PPAR signaling
Cell cycle
Hypertrophic cardiomyopathy
Thermogenesis
FoxO signaling pathway
Cardiac muscle contraction
Wnt signaling
Calcium signaling
Motor proteins
Dilated cardiomyopathy
Adrenergic signaling
GeneRatio
p.adjust
(E)
KEGG Pathway Enrichment - MTJ
Downregulated
Upregulated
TCA cycle
Glycolysis
TGF-beta signaling
Diabetic cardiomyopathy
FoxO signaling
Hippo signaling
Chemokine signaling
Autophagy
Oxidative phosphorylation
Pluripotency of stem cells
Spliceosome
AMPK signaling
Thermogenesis
mTOR signaling
Cardiac muscle contraction
Adrenergic signaling
Motor proteins
Apelin signaling
GeneRatio
p.adjust
(F)
KEGG Pathway Enrichment - NMJ
Downregulated
Upregulated
TCA cycle
Pyruvate metabolism
Glycolysis
Ubiquitin mediated proteolysis
Biosynthesis of amino acids
Diabetic cardiomyopathy
mTOR signaling
Motor proteins
MAPK signaling
GeneRatio
p.adjust
